# An Approach for Software-Intensive Business Innovation Based on Experimentation in Non-software-Intensive Companies

**DOI:** 10.1007/978-3-030-58858-8_1

**Published:** 2020-08-18

**Authors:** Kelson Silva, Eduardo Guerra, Jorge Melegati

**Affiliations:** 6 IT University of Copenhagen, Copenhagen, Denmark; 7 University of British Columbia, Vancouver, BC Canada; 8 Instituto Nacional de Pesquisas Espaciais, São José dos Campos, Brazil; 9 Free University of Bozen-Bolzano, Bolzano, Italy

**Keywords:** Innovation, Software-intensive business, Experimentation

## Abstract

Several companies whose businesses are not centered on technology might fail to innovate and get advantages over their competitors. For them, meaningful innovations are not necessarily related to the usage of new technologies but the optimization of some business process. In the literature, experimentation is described as an essential aspect of the innovation process. Although software engineering studies have explored experimentation, none has focused on software-intensive innovative projects in non-software-intensive companies, which consists of a contrast between the fast-changing environment in software-intensive to rigid structures in consolidated businesses. This paper proposes an experiment-oriented process to identify and implement innovation in this kind of company, including the roles involved in such processes. It has steps to identify business bottlenecks, search for solution alternatives, implement a fast and functional software proof of concept, create a plan for evolution, and migrate to a regular project to continue that idea. This paper also presents an evaluation of this process in a company focused on outsourced services, such as cleaning and maintenance. As a result, several internal procedures in a year were improved and received software support.

## Introduction

In an increasingly competitive world, the innovation-ability has to be developed in order to be able to survive in the long run, even in a non-high technological market. Although not being the focus of these companies, in many cases, the software may represent a way to non-software-intensive companies to innovate. Innovation is generally linked to a discontinuity in the marketing and/or technological process either in a macro (new to the world or industry) or micro-level (to the customer or the firm) [1].

In the literature, experimentation is a crucial activity to innovation [2]. Therefore, practices to promote experimentation are valuable to improve innovation. Based on experimentation, several practitioners oriented frameworks (e.g., Lean Startup [3]) have focused on innovation in companies developing a wide range of products. Recently, several studies proposed models to describe experimentation in software-intensive contexts (e.g., RIGHT [4] and HYPEX [5]). However, one context has received less attention: a non-software-intensive company pursues innovation through implementing a novel software system. There are several studies that investigate the usage of software development practices on companies in which software is not its primary focus [6, 7]. None of these studies clearly define the differences from this kind of company, but we assume that such a context may represent unique challenges due to the difference in culture that is significant for practices related to innovation. Consequently, this study will be guided by the following research question: **How can software-intensive business innovation be pursued in a non-software-intensive company?**

To answer the RQ, we proposed a framework to implement a software innovation process based on experimentation, including steps and roles. Given that the boundary between the framework implementation and the company operation is blurred, a case study is a proper research method choice. Such an option is standard while evaluating artifacts in software engineering research [8]. As an initial evaluation, we performed a case study in a Brazilian company called Guima ConSeCo. We present three internal innovation projects that were used as units of analysis: introduction of a recruitment portal, usage of a distance algorithm to reduce transportation costs, and a software-aided process for cleaning of hospital beds. Our results showed that the company succeeded in creating software-intensive innovations supporting the usefulness of the framework.

## Background and Related Work

The literature provides some examples of experimentation models. Olsson and Bosch [5] proposed the HYPEX (Hypothesis Experiment Data-driven Development). It consisted of six practices: generation of features that could be possibly valuable to the users; selection of highest priority features and creation of hypotheses about it; extraction of the so-called minimum viable feature or MVF: the smallest part that adds value to the customer; analysis of the difference between expected and actual behavior; in case of difference, development of hypotheses to explain it; and, finally, analysis of the hypotheses created and definition of what to do further.

Fagerholm et al. [4] proposed another model: RIGHT (Rapid Iterative value creation Gained through High-frequency Testing). It consists of cycles that start with the analysis of the learning obtained in previous cycles and the company’s business model and vision. Following, the team identifies and prioritizes hypotheses. Then, the team develops an MVP (Minimum Viable Product) or MVF to test a subset of hypotheses and update the instrumentation. Once the experiment is executed and its results are analyzed, the team reaches a decision-making stage. Based on what it learned, the team may persevere in the idea (implementing/optimizing, scheduling for deployment) or pivot it/change assumptions, etc.

Sveningson et al. [9] investigated the use of continuous experimentation in companies with low control of roadmap. The authors argued that there is a relationship among the control of the roadmap and the distance to the users to the use of continuous experimentation.

Melegati et al. [10] argued that these models follow a similar cycle consisted of the following steps: 1) identify, specify, and prioritize hypotheses, 2) design an experiment, 3) execute it, and 4) analyze its results. These results will lead to learning that will be used to feed the process by, if needed, updating the hypotheses.

Nevertheless, to the best of our knowledge, there is no systematic approach to perform software-intensive innovation in non-software intensive companies, highlighting the research gap we are diving into.

## Experiment-Driven Business-Oriented Innovation Approach

In this section, we present a proposed approach for software-intensive business innovation based on experimentation, including the roles needed. To describe it, we will detail each of the experimentation process steps identified by Melegati et al. [10] could be performed.

### Roles

In the context where a non-software-intensive company wants to innovate, the first thing that should be done is to form an innovation team. This group will be responsible for identifying the opportunities for improvement and for following the results and the initial implementation. This team should include a business specialist that understands the market where the company is included. Additionally, there should also be someone with good company knowledge, that knows and understands its current main problems and difficulties. Both roles might be played by the same person or not.

The team should also have technology experts who can propose and design new solutions, including elements for software support. The expertise should be on the design of software systems but not necessarily implementation since, for the project execution, another team should be assigned. Since the company’s primary focus is not technology, it might internally do not have an appropriate person for this role. In this case, it can be an external consultant or someone from a technology partner company. It is also advised to have someone with a research background to structure the projects and experiments to ensure they gather the appropriate data to evaluate the business goals’ suitability.

This innovation team does not need to be full-time because their members would not necessarily work on the projects, but they will identify and prioritize the innovation projects, evaluate the results, and make decisions. Because of that, the team should also have someone with the power to make decisions inside the company. The level of hierarchy required depends on the company, but there should be someone on this team that can assign for the innovation projects the necessary resources, especially to allocate the time of persons in key positions of the company that is important for a given project.

### Steps

The first step is the identification of hypotheses about a possible innovation. In this step, the innovation team has to identify opportunities for improvement. One kind of these opportunities can be related to the company’s recurrent problems in some process or area. As an example, it can be a process that is not fulfilling its goals or spending more resources than it should. The target process might also not be considered with problems, but it could have points that can be improved and optimized. It can also be an opportunity to expand the company business by developing a new product or expanding the market for an existing one. The identified opportunities are prioritized, and the innovation team should define what projects should be initiated.

Once a project is chosen, in the next step, a team should be assigned to design and perform an experiment. This team might include members from inside the innovation team, and also include others from outside, even from the target company, independent consultants, and partner company. It is essential to perform a feasibility check, verifying if the team has the necessary technical and business skills and knowledge to execute the project.

For each innovation project, it should be clear the scope and how much time the team has to work. Each project should be short, usually from one to three months. Due to the uncertainty of the results, it is better to fragment projects with a broader scope, defining at first a small one with a more limited scope and chain it with another defined based on the initial results. Usually, the output for each project can be either a critical answer to move on a relevant company issue or a functional proof-of-concept that can be implemented or evaluated even with some limitations.

In this step, the team assigned to work on the innovation project should search for solution alternatives that can solve the target problem. The solution can involve a change in the work procedure and the adoption of new techniques; however, it often involves the usage of the software that can support the adoption of the new approach. Before proposing the development of new software, it should be investigated if there is a product that can fulfill even partially those needs in the market and how they can be integrated with the existing solutions. After the possible solutions are evaluated, they are presented to the innovation team, giving their feedback and approval to proceed to an experimentation phase.

In the next step, the team implements a proof-of-concept to evaluate the proposed solution. It can be the installation of existing software or the creation of a prototype of a new one. Even when existing software is used, it is usually necessary for some development effort to integrate it with the existing applications and databases. The resulting products of this phase should be developed in small iterations and frequently delivered to get feedback from the company’s business side. Members of the innovation team should also follow these results.

When the innovation team judges that the solution was explored enough, it follows to the final step: analyze the results accomplished. Based on that, they make a final decision if the company is going to move forward in that direction or not. With a positive response, the project leaves the innovation team and become a regular company initiative. New related innovation projects might be created. However, the part already well established might now be managed as a regular project.

For various reasons that can be related to results, budget, or even focus, an innovation project might be abandoned. In this scenario, the innovation team should keep the knowledge obtained in the project in a way that can be used in the future, preserving valuable findings by applying Knowledge Management techniques. For instance, it is not rare for an idea that is not viable to be implemented in the present to be practicable later due to technological advances.

## Case Study

To evaluate the proposed approach, we performed a case study. This research approach has been used in software engineering literature to the research purpose of improvement [8], like our study.

The proposed approach was applied in a Brazilian company called Guima-ConSeCo, focused on outsourcing services, such as cleaning and maintenance engineering. During our study, the company developed three innovation projects using the proposed approach. Each one is a unit of analysis. Data collection consisted of participant observation and the analysis of the project’s documentation.

The company currently has around 10.000 employees and provides services in several public and private facilities. The company has plans to grow in size, and its managers feel the need to innovate in certain aspects to make the company more competitive and enable sustainable growth.

The company aims to take advantage of a Brazilian incentive law for innovation^1^. Several countries also have similar laws to incentivize companies to develop innovation. In Brazil, the company can use a small percentage of its taxes to invest in this kind of project. To get the benefit, the company should document and submit the project to the authorities.

The innovation team was composed of members of a partner technology company named Jetsoft. The third-party company was responsible for proposing and implementing this process in the target company. Jetsoft started performing a mapping study of all the business processes from the target company, identifying problems and potential points for improvement. The team works with a tool named Genexus [11], which allows fast development and generates code to several platforms such as Java and .NET. The speed provided by this tool was important for fast prototyping the solutions.

In all projects described, granular experiments happened, proposing solutions to parts of the problem and validating them quickly. In many cases, the solutions were discarded for not passing in an initial evaluation. The approved solutions were put in practice on a small scale, being refined until being mature enough for being deployed to production.

### Units of Analysis

**Recruitment Portal:** As a company that manages outsourcing services for a high number of employees, human resources is a crucial area, especially recruitment. The motivation for this innovation project was that a high number of candidates delivered their resume in a paper, which was generating much manual work, and nevertheless, many of them were not being processed. This innovation project proposed the creation of a recruitment portal were the candidates can add their resume. Additionally, an internal application was developed to allow a search through this resume database. The initial experiments generated a working prototype, and further, this solution was evolved to become a company product. After two months of the start of this innovation project, an initial version was online. As a result, the human resources manual work reduced, and now the company has a more suitable tool to search for potential job candidates for a given position. Currently, the application database has around 18k resumes.

**Distance Algorithm:** The company manages employees working in different areas of the city. Some of them worked in places far from their home, even having alternatives close to where they live. This mismatch generates costs for the company like transportation, possible delays due to traffic, and a drop in employee’s quality of life. This project proposed an algorithm to calculate the distance between the workplace and the candidate’s address to be used in the recruitment process. An experiment was performed executing the algorithm direct on the data. After its validation, it was integrated into the recruitment portal mentioned in the previous section. The automation of this distance calculation is currently being used in the recruitment process, and it is considered, as a next step, to be used for workplace optimization of current employees.

**Cleaning of Hospital Beds:** The cleaning of hospitals is one of the services provided by the target company. Several problems occurred in this service because the contracting hospital supervisor often evaluated the service after an extended period, which can lead to divergences. An opportunity for improvement was detected with the proposal of a tool that can be used to manage the requests for cleaning and supervision, allowing the capture of images that can register the service performed by the employee and problems found by the supervisor. The company also considered that with this data, it would be possible to evaluate the efficiency from different angles and perform actions to optimize the work. In this innovation project, the first step was to search for products that could fulfill those needs. Some potential matches were found, and meetings with their companies were made to get knowledge about them. It was found that two of them could partially fulfill the requirements, the central problem being the integration with the current systems. An experiment with these two different solutions was done in different clients, and both are currently being used in production after adjustments from the providers.

## Discussion and Conclusions

In each analysis unit, it was possible to observe that the company successfully implemented an innovation. Such achievements were reached either by implementing prototypes or by selecting a solution already existent in the market and then experimenting and evaluating the results. Looking to the case considering the target research question: “**How can software-intensive business innovation be pursued in a non-software-intensive company?**”, we can conclude that the proposed approach can be a valid way for that. The target company was able to innovate in some of its critical areas for its business, and intend to keep applying this process continuously.

We considered the focus on business problems as one of the critical success factors of this approach. Innovation can be seen wrongly as the usage of breaking edge technologies, however, with the focus on business, the use of simpler software technology could solve the problem if it is appropriate for the demands. Another critical point for the success in the studied case was the partnership of the non-software-intensive company with a company specialized in business process mapping and software development. That created a synergy where each one focuses on its field of specialization but collaborates to reach a common goal.

Although the innovation projects described do not represent anything new to the world, they can be described as micro-level innovations [1]. That is, the solutions proposed represented novelty at the firm level, and its development consisted of a challenge to the company. The novelty of the proposed approach when compared to previous experimentation models and Lean Startup is its focus on the dichotomy software-intensive innovation in a non-software-intensive context. Such target allowed the description of specific roles and activities. It is important to highlight that this work is still in progress and our goal is to tackle the lack of operationalization of Lean Startup, as previously recognized in the literature (e.g., [12]).

The proposed approach starts with identifying problems and opportunities for improvement, creating a team that identifies potential solutions, and validates them through experiments. It was evaluated through a case study in a company focused on outsourcing of cleaning and maintenance. The results of the case study imply that the proposed approach is feasible and suitable since it helped a non-software-intensive business company pursue innovation based on software-intensive business solutions.

As future work, we will collect more data in the presented case study through interviews with different actors involved in the process. The goal is to acquire information about the main difficulties and try to identify other lessons learned. We intend to implement the same process in other companies, intending to generalize practices, and identify difficulties that can arise in a different context. Another future study could identify the differences and peculiarities from non-software-intensive companies relevant for innovation projects.

## Footnotes

1 Law No 10.973 2nd December 2004 - http://www.planalto.gov.br/ccivil_03/_ato2004-2006/2004/lei/l10.973.htm.
